# Urinary Biomarkers in Screening for the Usual Intake of Fruit and Vegetables, and Sodium, Potassium, and the Sodium-to-Potassium Ratio: Required Number and Accuracy of Measurements

**DOI:** 10.3390/nu16030442

**Published:** 2024-02-01

**Authors:** Aoi Suzuki, Ribeka Takachi, Junko Ishihara, Sachiko Maruya, Yuri Ishii, Kumiko Kito, Kazutoshi Nakamura, Junta Tanaka, Taiki Yamaji, Hiroyasu Iso, Motoki Iwasaki, Shoichiro Tsugane, Norie Sawada

**Affiliations:** 1 Department of Food Science and Nutrition, Nara Women’s University Graduate School of Humanities and Sciences, Kitauoyahigashimachi, Nara-shi 630-8506, Nara, Japan; waa_suzuki@cc.nara-wu.ac.jp (A.S.); s-maruya@cc.nara-wu.ac.jp (S.M.); 2 Graduate School of Environmental Health, Azabu University, 1-17-71 Fuchinobe, Chuo-ku, Sagamihara-shi 252-5201, Kanagawa, Japan; j-ishihara@azabu-u.ac.jp; 3 Division of Cohort Research, National Cancer Center Institute for Cancer Control, 5-1-1 Tsukiji, Chuo-ku, Tokyo 104-0045, Japan; yurishii@ncc.go.jp (Y.I.); kitou@azabu-u.ac.jp (K.K.); moiwasak@ncc.go.jp (M.I.); stsugane@ncc.go.jp (S.T.); nsawada@ncc.go.jp (N.S.); 4 Division of Preventive Medicine, Niigata University Graduate School of Medical and Dental Sciences, 1-757 Asahimachidori, Niigata 951-8510, Japan; kazun@med.niigata-u.ac.jp; 5 Department of Health Promotion Medicine, Niigata University Graduate School of Medical and Dental Sciences, 1-757 Asahimachidori, Niigata 951-8510, Japan; juntatnk@med.niigata-u.ac.jp; 6 Division of Epidemiology, National Cancer Center Institute for Cancer Control, 5-1-1 Tsukiji, Chuo-ku, Tokyo 104-0045, Japan; tyamaji@ncc.go.jp; 7 Institute for Global Health Policy Research, National Center for Global Health and Medicine, 1-21-1 Toyama, Shinjuku-ku, Tokyo 162-8655, Japan; hiso@it.ncgm.go.jp; 8 Graduate School of Public Health, International University of Health and Welfare, 4-1-26 Akasaka, Minato-ku, Tokyo 107-8402, Japan

**Keywords:** screening, fruit and vegetable intake, biomarker, receiver-operating characteristic analysis, within- and between- individual variation

## Abstract

Because of within-individual variation, surveys to estimate an individual’s usual food intake must be conducted over many days, in general. Here, using non-invasive biomarkers, we examined the number of measurements required to screen for the usual intake of fruit and vegetables, in addition to sodium, potassium, and the sodium-to-potassium (Na/K) ratio. Participants were 202 subjects aged 40–74 years from five areas of Japan who completed weighed food records (WFR) and five 24-hour urinary collections (24-h UCs) between 2012 and 2013. The number of 24-h UCs required to screen for intake that deviated from guidelines estimated by the WFR and their accuracies were assessed by the area under the curve (AUC) in a receiver-operating characteristics (ROC) analysis. The single urinary excretion of sodium, potassium, and the Na/K ratio showed moderate performance (AUC value: >0.7) in discriminating deviations from their criteria by respective intake based on the WFR. Urinary potassium excretion also showed moderate performance (AUC value: >0.7) in estimating the intake of vegetables but could not be used to estimate fruit intake even after five collections. The non-invasive measurement of biomarkers in a single 24-h UC showed moderate performance in screening the usual intake of vegetables, as measured based on the 12-day WFR, as well as of sodium, potassium, and the Na/K ratio.

## 1. Introduction

Given findings that a high intake of sodium and low intake of fruit and vegetables are major adverse dietary factors for death and disability-adjusted life years (DALYs) [1], the quantitative assessment of the adherence of an individual’s usual intake of sodium, fruits, and vegetables to guidelines defined by the WHO [2,3] or individual countries may be particularly important.

Fruit and vegetable intake is better measured using objective methods, because measurements using subjective methods tend to result in overestimation [4]. While serum carotenoids and plasma vitamin C are used as biomarkers of fruit and vegetable intake [5,6], their use is limited by the difficulties they present in obtaining total intake [7], the lack of quantification [8], and invasiveness. Skin carotenoids (carotenoids in sebum of the palm) are non-invasive biomarkers that can be measured via spectroscopy technologies, including resonance Raman spectroscopy (RRS), reflection spectroscopy (RS), and spectrophotometers [9]. However, studies of the validity of these methods have been limited to correlations with blood carotenoid concentrations or the self-reported intake of fruit and vegetables or carotenoids. Moreover, urinary potassium has been rarely used as a biomarker of fruit and vegetable intake [6], notwithstanding the substantial contribution of fruits and vegetables to potassium intake [10,11,12]. With regard to sodium, the estimation of sodium intake based on food records or 24-hour dietary recall is considered relatively inaccurate [13]. Sodium intake can be estimated based on urinary excretion, but since this reflects recent intake only [14], which may vary widely from day to day, usual intake is better determined using multiple 24-hour urinary samples [15,16,17]. To be effective, screening tools for both potassium and sodium should be able to quantitatively assess adherence to usual intake. Their implementation is particularly advantageous when they are primarily non-invasive and require less frequent measurements or a less frequent need to weigh and record all eaten foods.

Against this background, we examined the number of 24-h urinary collections (24-h UCs) required to screen for guideline deviations in intake determined using 12-day weighed food records (12-d WFR) and their accuracy. In addition, we also examined the degrees of error required to assess individual intakes based on 12-d WFR and the variability of estimates obtained from the WFR compared with those using the 24-h UC.

## 2. Materials and Methods

### 2.1. Study Setting and Participants

The study was conducted in five areas included under the Japan Public Health Center-based Prospective Study for Next Generation (JPHC-NEXT) protocol (Yokote, Saku, Chikusei, Murakami, and Uonuma). Details of the study design and methods of data collection have been described elsewhere [18,19,20]. Of 255 participants at the beginning of the study, 202 participants (80 men and 122 women) aged 40–74 years without missing data on creatinine who completed a 12-d WFR and five 24-h UCs were included in the analysis.

This study was carried out accordance with the Declaration of Helsinki and approved by the Institutional Review Boards of the National Cancer Center, Tokyo, Japan, and of all other collaborating research institutions, including the Ethics Review Committee of Nara Women’s University. All participants provided written informed consent to participate in the study.

### 2.2. Data Collection

The 12-d WFR and five 24-h UCs were conducted between November 2012 and December 2013. WFRs were conducted for three consecutive days over four seasons at approximately 3-month intervals. The 24-h UCs were collected on the last day of each 3-day WFR and one year after the start of the survey. Information on age and anthropometric data was obtained using a self-administered questionnaire.

### 2.3. 12-d WFR

Each 3-day WFR was conducted for three consecutive days, consisting of two weekdays and one weekend day in each of the four seasons. Food portions were measured by each participant during meal preparation using a supplied precise portable digital cooking scale (Tanita Co., Ltd., Tokyo, Japan) and measuring spoons and cups. For meals purchased or consumed outside the home, the participants were instructed to record the approximate quantity of all foods in the meal and/or the name of the product and company. Dieticians checked food records with the participants the day after each of the 3-day WFR on site in each study area. The intake of total sodium and potassium was calculated using the Standard Tables of Food Composition in Japan 2010 (FCT) [21]. Fruit and vegetable intake was defined according to food groups in the FCT. Potassium intake for the sodium-to-potassium (Na/K) ratio was adjusted to the urinary excretion level by dividing by 1.3 [12,22]. The Na/K ratio was calculated as a molar ratio.

### 2.4. 24-h UC

The participants collected urine samples using a portable urine measurement device (sumius U-Container, Sumimoto Bakelite Co., Ltd., Tokyo, Japan), which obtains a 1/50 portion of all collected urine. A single urine collection error (e.g., forgetting to conduct the sampling or spillage out of the container) was corrected using the mean value based on the individual’s collected urine volumes and recorded number of error-free collections. Participants who had two or more errors in any one of a total of five urine collections during the study period were excluded from analysis. The 24-h urinary sodium and potassium excretion were calculated using the following formulas: 24-h urinary sodium excretion (mg) = obtained volume of urinary excretion (mL) × 50/1000 × urinary sodium concentration (mEq/L) × 23; and 24-h urinary potassium excretion (mg) = obtained volume of urinary excretion (mL) × 50/1000 × urinary potassium concentration (mEq/L) × 39. Furthermore, potassium excretion was adjusted to the intake level by multiplying by 1.3 [12,22]. The Na/K ratio was calculated as a molar ratio. Additionally, to examine the usefulness of urinary concentrations, we used 24-h urinary sodium, potassium, and creatinine concentrations instead of second-morning voiding urine for Kawasaki equations [23], which were originally proposed to estimate the 24-h urinary sodium and potassium excretion from the second voiding of urine in the morning. Since the Na/K ratio can be calculated based on the concentration, the Kawasaki equations are not required and were therefore not calculated.

### 2.5. Statistical Analysis

To examine the accuracy of urinary sodium, potassium, and Na/K ratio values obtained from the 24-h UCs in discriminating their usual intake, the area under the curve (AUC) and its 95% confidence intervals (CIs) were calculated via receiver-operating characteristic (ROC) analysis using the mean values of the 12-d WFR as the reference standard. Sensitivity was defined as the probability of discriminating whether a person whose intake deviated from the criterion based on the 12-d WFR also deviated by 24-h UC. Specificity was defined as the probability of discriminating whether a person whose intake did not deviate from the criterion by the 12-d WFR also did not deviate based on the 24-h UC. Similarly, to screen the intake of fruit and vegetables, the accuracy of urinary potassium was examined. As criteria, the tentative dietary goals for preventing lifestyle-related diseases (DG) in Dietary Reference Intakes for Japanese (2020) [24] were used for sodium (<7.5 g for men and <6.5 g for women, salt equivalents) and potassium (≥3000 mg for men and ≥2600 mg for women). For the Na/K ratio, previous studies considered a tentative target value of a molar ratio < 2.0 [25,26]; however, because few participants met this criterion, the median value of the 12-d WFR was also used; ≥350 g per day for vegetable intake and ≥100 g per day for fruit intake were used as criteria, as defined in the Basic Direction for Comprehensive Implementation of National Health Promotion (Health Japan 21, the Second Term) [27]. Screening was defined as useful if the AUC was >0.7 and the lower limit of the 95% CI was >0.5 [28]. The optimal cut-off value was determined based on the Youden Index and the minimum distance between the upper left point and each point on the ROC curve. The Youden Index and distance to the corner were calculated using these formulas: Youden Index = sensitivity + specificity − 1, and distance to corner = (1 − sensitivity)^2^ + (1 − specificity)^2^. Sensitivity and specificity at the optimal cut-off value were also selected.

Five 24-h UCs for each participant were randomly arranged, and the cumulative mean values from one to five collections were calculated for each. Using these values, we examined the number of 24-h UCs required for screening. Furthermore, we conducted sub-analyses to examine the usefulness of screening using values other than the aforementioned values as criteria, as follows: for every 1 g between <7 and <15 g (salt equivalent) for sodium, for every 500 mg between ≥2000 and ≥4000 mg for potassium, for every 0.5 units between <2.0 and <4.5 units for the Na/K ratio, and for every 50 g between ≥50 and ≥250 g and between ≥200 and ≥550 g for fruit and vegetables, respectively. Analyses that discriminated the combined intake of fruit and vegetables were also conducted. The criteria were defined as ≥250 to ≥800 g in 50 g increments.

The degrees of error in the 12-d WFR used as a reference to screen intake were estimated using the following analysis. Within-individual variance and between-individual variance in sodium, potassium, and the Na/K ratio were calculated using the proc varcomp procedure (SAS version 9.4 software, SAS Institute Inc., Care, NC, USA) [29] for the estimated values obtained from the WFR, 24-h urinary excretion, 24-h urinary concentrations, and estimated excretion values using Kawasaki equations, respectively. Values of fruit and vegetable intake based on the WFR were also calculated. Coefficients of within-individual variation (CV_w_) and of between-individual variation (CV_b_) were calculated using the following formulas: CV_w_ (%) = {(within-individual variance)^0.5^/mean} × 100; and CV_b_ (%) = {(between-individual variance)^0.5^/mean} × 100. In this study, untransformed data were used to calculate CV_w_ and CV_b_, since previous studies [30,31] showed several problems with transformed data, namely that estimates based upon transformed data were difficult to interpret meaningfully, back-transformation may introduce considerable bias to the variance estimates, and normality was not improved by log-transformation. The number of survey days required to estimate mean intake within a specified percentage deviation (meaning 95% CIs) of the individual mean from the usual (“true”) mean value based on the CV_w_ was calculated using the following formula [14]: *n* = (Z_α_ × CV_w_/D)^2^, where *n* = the number of days required to estimate per person, Z_α_ = 1.96, and D = a certain degree of error as a percentage of true usual intake. The number of days required was determined assuming errors of ±5% (10%), ±10% (20%), and ±15% (30%), respectively. All analyses were performed using SAS version 9.4 (SAS Institute Inc., Care, NC, USA).

## 3. Results

Table 1 shows the characteristics of participants. The intake of sodium and potassium and the Na/K ratio were greater in men than in women. The median fruit intake based on individual means with the 12-d WFR was greater in women than in men, whereas vegetable intake was similar between the sexes.

The required number of 24-h UCs and their accuracy in screening for deviations from the guideline intake of sodium and potassium based on the 12-d WFR are shown in Table 2. Both sodium and potassium could be discriminated by a single 24-h urinary excretion measurement with AUC values > 0.7. Accuracies did not greatly change even when the cumulative number of measurements was increased. Concentrations were not useful in discriminating based on the guideline for either sodium or potassium. Most of the excretion values estimated using Kawasaki equations for sodium and potassium were as accurate as excretions. A single 24-h urinary Na/K ratio was also useful, with an AUC value > 0.7 when the median value of the 12-d WFR was used as the criterion (Table 2). A similar result was obtained when <2.0 was used as the criterion, albeit few participants actually met this criterion.

Spearman’s correlation coefficient between a single randomly selected 24-h urinary potassium excretion measurement and vegetable intake based on the 12-d WFR was 0.50 for men and 0.48 for women. Respective coefficients for fruit intake were 0.25 and 0.35. Regarding vegetables, when ≥350 g was used as the criterion, screening was useful for the 24-h urinary potassium excretion, regardless of sex (Table 3), but not with the concentration. All potassium excretion values estimated using Kawasaki equations were as accurate as the 24-h urinary potassium excretion. However, 24-h urinary potassium excretion was not useful for ≥100 g of fruit as a criterion regardless of sex or the cumulative number of collections (Table 3).

In addition, single urinary sodium excretion was able to discriminate <7.0 to <12.0 g in 1 g increments for salt intake (Table S1). Moreover, a single 24-h urinary potassium excretion was able to discriminate ≥2000 to ≥3500 mg in 500 mg increments for potassium intake (Table S1). The Na/K ratio in a single urine collection could discriminate <2.0 to <4.5 in 0.5-unit increments of the Na/K intake ratio (Table S1). A single 24-h urinary potassium excretion was also able to discriminate ≥400 g and ≥500 g for vegetable intake and ≥300 to ≥450 g and ≥550 to ≥650 g for the combined intake of fruit and vegetables in 50 g increments, regardless of sex (Table S2). However, fruit intake could not be discriminated from a single 24-h urinary potassium excretion in any criteria in either sex (Table S2).

The coefficients of variation in estimates and the number of days required to estimate an individual’s usual mean intake with a certain degree of error as a percentage of usual intake are shown in Table 4. CV_w_ and CV_b_ of the 12-d WFR were almost the same as for the five 24-h UCs for sodium, potassium, and the Na/K ratio. The CV_w_ of vegetables was greater than those of other nutrients, while the CV_w_ of fruit was even greater. WFR of 8, 6, and 10 days, respectively, was needed to estimate the 95% CI of the usual mean intake of sodium, potassium, or the Na/K ratio within ±10%, regardless of sex. Similarly to these, based on 24-h urinary excretion, the corresponding number of days required was 10, 7, and 10 days for men and 9, 7, and 8 days for women, respectively. In contrast, the intake of fruit and vegetables needed more days of WFRs for these evaluations (vegetables, 22 and 18 days for men and women, respectively; fruit, 115 and 72 days for men and women, respectively). As a result, for the 12-d WFR that we used as a reference to screen intake, the intake of sodium and potassium and the Na/K ratio were evaluated with 95% CIs within ±5% to ±10% of an individual’s usual mean intake. In the WFR, the number of days required to estimate the error (95% CI) to within ±5% to ±10% of an individual’s mean intake for sodium, potassium, and the Na/K ratio was similar to that obtained with 24-h UCs. Vegetable and fruit intake based on the 12-d WFR were evaluated with 95% CIs to be within ±10% to ±15% and wider than ±15% of an individual’s mean of the usual intake, respectively.

## 4. Discussion

We found that the evaluated intake of sodium, potassium, and the Na/K ratio from a 12-d WFR with 95% CIs was within ±5% to ±10% of an individual’s usual mean intake, similar to intakes evaluated based on the same number of 24-h UCs. The intake of sodium and potassium and the Na/K ratio, based on the 12-d WFR, could be discriminated from on the single 24-h urinary excretion for almost all criteria. Vegetable intake, but not fruit intake, could be discriminated using single 24-h urinary potassium excretion for some criteria.

Using a 12-d WFR in men and women aged 45–77 years, Ogawa et al. [29] reported that eight days of WFRs were required to estimate the usual mean sodium intake with a 95% CI within ±10% for both men and women. They also showed that seven days and five days of WFR were required to estimate the usual mean potassium intake with a 95% CI within ±10% for men and women, respectively. Fukumoto et al. [30] used a 16-d WFR in men and women aged 50–69 years to show that 11 days were needed to assess the usual mean intake of sodium with a 95% CI within ±10% based on the WFR for both men and women. To assess the usual mean intake of potassium with a 95% CI within ±10%, five days and seven days were required for men and women, respectively. These results are consistent with our present finding that an individual’s usual mean sodium and potassium intake can be estimated based on the 12-d WFR with a 95% CI within ±10%. In addition, Ogawa et al. [29] also reported that an assessment of an individual’s usual vegetable intake with a 95% CI within ±10% required 18 days and 16 days of WFRs for men and women, respectively. They also reported that an assessment of an individual’s usual fruit intake with a 95% CI within ±10% required 140 days for men and 64 days for women. These values are consistent with the trend in our present study, specifically that assessing an individual’s usual mean intake of vegetables requires more days than the number required to assess the intake of individual nutrients and that assessing fruit intake requires even more days. To our knowledge, the few previous studies on the CV_w_ and CV_b_ of sodium or potassium measured based on 24-h urine [32,33,34] used two or four 24-h UCs. Our results were based on a larger number of 24-h UCs than these previous reports and were consistent with them.

Previously, the relationship between sodium intake based on the WFR and 24-h urinary sodium excretion was examined from the correlation and mean difference [34,35]. Further, one study [18] discriminated based on WFR using the 24-h UC as a reference standard. To our knowledge, however, no previous study has quantitatively investigated intake using biomarkers in comparison with the WFR as a reference standard.

In the present study, urinary potassium could be used to discriminate vegetable intake, but not fruit intake. This might be attributable to the difference in the percentage contribution to total potassium intake among food groups; the largest was for vegetables (30.6%), whereas fruits were only the fourth largest (7.3%). This is likely consistent with the National Health and Nutrition Survey in Japan in 2013 [36], which measured that at 22.8% and 8.8% for vegetables and fruit, respectively. This relatively small contribution of fruit to potassium intake may be one reason why urinary potassium excretion could not be used to determine fruit intake. A second reason for the inability to discriminate fruit intake by urinary potassium excretion may be that the 12-d WFR, which was used as a reference, may not have adequately reflected the usual intake because of its large CV_w_.

Fujioka et al. [37] reported a dose–response relationship between intake of 25, 50, 100, 200, 300, 400, or 500 µmol of glucobrassicin (based on a Brussels sprouts- and/or cabbage- feeding session), which is abundant in cruciferous vegetables, and urinary 3,3′-diindolymethane (DIM), which is one of its metabolites. The correlation was relatively high, at R^2^ = 0.68, albeit they considered cruciferous vegetables only. We considered that urinary potassium, as a major nutrient, is more convenient for screening total vegetable intake. However, Krogholm et al. [38] reported no differences in urinary potassium excretion among groups with feeding interventions of 0, 300, and 600 g of fruit and vegetables using urine samples from the day before and the day of the intervention. Because urinary biomarkers are thought to be reflected by the day of intake and during the week thereafter [7], it is possible that intake was not adequately reflected in the urine in their study. In our present study, we showed that usual total vegetable intake can be screened by urinary potassium excretion using four of five 24-h UCs conducted on the last day of each 3-d WFR.

With regard to fruit, we speculate that a combination of several biomarkers may be suitable for determining intake. McNamara et al. [39] developed a multi-biomarker panel using spot urine for fruit intake and examined its agreement with intake. The fruit intake intervention consisted of low, medium, and high portions of provided fruit, namely 50, 100, and 300 g for apples and 80, 160, and 320 g for oranges, respectively, in 160 men and women aged 18–60 years for four consecutive days each week over five weeks. They collected fasting first-void urine after an overnight 12-h fast at the end of each study week. Proline betaine, hippurate, and xylose were selected based on a metabolomic analysis of urine, and a multi-biomarker panel was created by summing the values of the three biomarkers per participant. Cut-off values of ≤ 4.766, 4.766–5.976, and >5.976 μM/mOms/kg were defined for the multi-biomarker panel for fruit intake of <100, 101−160, and >160 g. They then used the total fruit intake obtained from semi-weighed food records for four consecutive days and fasting first-void urine collected at or as close to the end of the food record as possible for 546 men and women aged 18–90 years and examined agreement among <100, 101−160, and >160 g of fruit intake and ≤4.766, 4.766–5.976, and >5.976 μM/mOms/kg of the multi-biomarker panel. The results showed good agreement. The biomarkers used to estimate foods or food groups should be specific biomarkers [7,8]. The combination of values of the three biomarkers selected by McNamara et al. may have been specific for fruit intake, but their methods were nevertheless not simple. Furthermore, fruit and vegetable intake is subject to seasonal variation. The data they used to develop their multi-biomarker panel were based on an intake intervention of apples and oranges for four consecutive days each week over five weeks, which accordingly excluded any consideration of seasonal variation. In contrast, the 12-d WFR used as a reference standard in our present study collected data in each of the four seasons of a single year and could therefore be used to evaluate intake with the consideration of seasonal variation.

With regard to other non-invasive biomarkers for the individual intake of vegetables or fruit, Radtke et al. [9] reviewed the accuracy of skin carotenoid measurements using spectroscopy technologies, such as RRS, RS, and spectrophotometers, which had been examined by calculating the correlation between blood carotenoids (serum and plasma) or dietary carotenoids and fruit and vegetable intake, as estimated using self-reported methods. The correlation coefficients for skin carotenoids were reported to range from weak to strong (0.39 to 0.81) for blood carotenoids, from weak to moderate (0.41 to 0.60) for dietary carotenoids, and from weak to moderate (0.22 to 0.47) for fruit and vegetable intake. In that review [9], the studies that examined the association between skin carotenoids and both blood carotenoids and dietary intake showed moderate-to-strong correlation coefficients with blood carotenoids (0.62–0.79), whereas the correlation coefficients with dietary intake were all lower than those with blood carotenoids. In our present study, the correlation coefficient between single 24-h urinary potassium excretion and vegetable intake from the 12-d WFR was slightly higher (0.48 to 0.50) than those between skin carotenoids and the intake of fruit and vegetables (0.22 to 0.47) in the review. Additionally, to our knowledge, no previous study has examined the accuracy of screening the individual consumption of fruit and vegetables based on skin carotenoids.

This study has some limitations. First, the urine used in this study was not spot urine. The accuracy of estimation using the Kawasaki equation, which originally used spot urine, was not properly evaluated because we used the 24-h UC. We consider that the 24-h UC probably overestimates the accuracy of the evaluation compared with spot urine, and further examination using spot urine is required. Second, the participants were skewed toward middle-aged and elderly adults. It has been reported that the CV_w_ is smaller in elderly people than in younger people [30]. Accordingly, the CV_w_ obtained from our participants may also have been smaller than those of younger people. It is possible that the accuracy of the discrimination may have been overestimated due to our measured values—obtained with a 12-d WFR—more closely reflecting usual intake, given that the screening accuracy of fruit intake was low with a larger CV_w_ than that seen with the others.

## 5. Conclusions

In conclusion, this study suggests that deviations from the criteria for sodium and potassium intake and the Na/K ratio established using a 12-d WFR could be differentiated using a single 24-h UC. Vegetable intake could also be differentiated based on a single 24-h urinary potassium excretion. In contrast, fruit intake could not be determined using the cumulative average of multiple urinary potassium excretions.

## Figures and Tables

**Table 1:** Characteristics of participants.

	Men (*n* = 80)		Women (*n* = 122)	
	Median	Interquartile Range	Median	Interquartile Range
Age (years)	59	52–64	58	51–64
Body weight (kg)	67.0	61.0–71.5	55.0	50.0–59.0
Body height (cm)	167.5	163.0–173.0	156.0	152.0–160.0
BMI (kg/m^2^)	23.8	22.2–25.2	22.4	20.8–24.1
Sodium intake (mg, WFR)	4424	3772–5267	3694	3155–4218
Potassium intake (mg, WFR)	3236	2588–3752	2934	2394–3454
Na/K ratio in intake (mol/mol, WFR)	3.3	2.7–3.7	2.9	2.4–3.3
Fruit intake (g)	78	36–151	131	73–191
Vegetable intake (g)	336	241–452	332	257–429

BMI, body mass index; WFR, weighed food record.

**Table 2:** AUC (95% CI) of the ROC curve of 24-hour urinary sodium, potassium, and the Na/K ratio to detect deviation from dietary intake measured based on the 12-day WFR.

		Men							Women						
		AUC	95% CI	CO ^a^	Se	Spe	YI	DC	AUC	95% CI	CO ^a^	Se	Spe	YI	DC
Sodium		Criterion < 7.5 g (*n* ^b^ = 75/80)							Criterion < 6.5 g (*n* ^b^ = 115/122)						
1 time	Excretion	0.77	0.63–0.92	3621	0.67	0.80	0.47	0.39	0.84	0.74–0.94	3074	0.77	0.86	0.62	0.27
	Concentration	0.55	0.21–0.89	66	0.92	0.40	0.32	0.61	0.69	0.44–0.93	95	0.62	0.86	0.47	0.41
	Kawasaki	0.72	0.52–0.91	4663	0.67	0.80	0.47	0.39	0.89	0.82–0.96	4184	0.84	0.86	0.70	0.21
2 times	Excretion	0.84	0.71–0.97	3347	0.87	0.80	0.67	0.24	0.88	0.82–0.94	2889	0.85	0.86	0.71	0.21
	Concentration	0.66	0.32–1.00	83	0.88	0.60	0.48	0.42	0.57	0.34–0.80	115	0.43	0.86	0.28	0.59
	Kawasaki	0.74	0.44–1.00	4638	0.72	0.80	0.52	0.34	0.90	0.84–0.96	4162	0.84	0.86	0.70	0.21
3 times	Excretion	0.85	0.69–1.00	3327	0.89	0.80	0.69	0.23	0.88	0.80–0.96	2990	0.83	0.86	0.69	0.22
	Concentration	0.64	0.28–1.00	82	0.87	0.60	0.47	0.42	0.58	0.33–0.82	96	0.60	0.71	0.31	0.49
	Kawasaki	0.78	0.52–1.00	4579	0.84	0.80	0.64	0.26	0.95	0.90–0.99	4087	0.91	0.86	0.77	0.17
4 times	Excretion	0.80	0.65–0.95	3549	0.83	0.80	0.63	0.26	0.83	0.71–0.95	3370	0.73	0.86	0.59	0.31
	Concentration	0.56	0.19–0.92	72	0.96	0.40	0.36	0.60	0.54	0.31–0.77	107	0.46	0.71	0.18	0.61
	Kawasaki	0.74	0.50–0.98	4665	0.85	0.80	0.65	0.25	0.89	0.80–0.97	4305	0.83	0.86	0.68	0.23
5 times	Excretion	0.76	0.59–0.93	4215	0.69	0.80	0.49	0.37	0.87	0.77–0.96	3265	0.79	0.86	0.65	0.25
	Concentration	0.54	0.24–0.85	89	0.81	0.40	0.21	0.63	0.48	0.25–0.72	85	0.28	0.86	0.14	0.74
	Kawasaki	0.71	0.44–0.98	4899	0.75	0.80	0.55	0.32	0.92	0.85–1.00	4217	0.92	0.86	0.78	0.16
Potassium		Criterion ≥ 3000 mg (*n* ^b^ = 34/80)							Criterion ≥ 2600 mg (*n* ^b^ = 40/122)						
1 time	Excretion ^c^	0.77	0.67–0.88	2814	0.74	0.78	0.52	0.34	0.76	0.67–0.84	3146	0.88	0.56	0.44	0.46
	Concentration	0.60	0.46–0.73	30.2	0.56	0.67	0.23	0.55	0.52	0.41–0.63	33.3	0.40	0.70	0.10	0.67
	Kawasaki ^c^	0.73	0.62–0.84	2734	0.71	0.72	0.42	0.41	0.73	0.63–0.82	2560	0.55	0.79	0.34	0.50
2 times	Excretion ^c^	0.82	0.73–0.91	3027	0.85	0.70	0.55	0.34	0.72	0.63–0.82	2605	0.60	0.76	0.36	0.47
	Concentration	0.62	0.49–0.75	33.1	0.62	0.63	0.25	0.53	0.50	0.39–0.60	46.5	0.83	0.27	0.09	0.75
	Kawasaki ^c^	0.76	0.66–0.87	2694	0.74	0.76	0.50	0.36	0.72	0.63–0.82	2704	0.75	0.63	0.38	0.44
3 times	Excretion ^c^	0.81	0.72–0.90	2811	0.76	0.74	0.50	0.35	0.78	0.68–0.87	2654	0.73	0.72	0.44	0.39
	Concentration	0.60	0.48–0.73	29.1	0.44	0.83	0.27	0.59	0.50	0.39–0.61	45.7	0.90	0.26	0.16	0.75
	Kawasaki ^c^	0.76	0.66–0.86	2681	0.65	0.78	0.43	0.41	0.78	0.68–0.87	2683	0.75	0.70	0.45	0.39
4 times	Excretion ^c^	0.81	0.71–0.90	3002	0.82	0.70	0.52	0.35	0.79	0.70–0.87	2653	0.70	0.73	0.43	0.40
	Concentration	0.58	0.45–0.72	28.7	0.44	0.78	0.22	0.60	0.50	0.38–0.61	39.2	0.65	0.41	0.06	0.68
	Kawasaki ^c^	0.76	0.65–0.86	2793	0.79	0.67	0.47	0.39	0.78	0.68–0.87	2488	0.58	0.88	0.45	0.44
5 times	Excretion ^c^	0.81	0.72–0.90	3024	0.88	0.63	0.51	0.39	0.79	0.70–0.88	2658	0.65	0.78	0.43	0.41
	Concentration	0.57	0.44–0.70	29.4	0.44	0.78	0.22	0.60	0.49	0.38–0.60	39.6	0.68	0.41	0.09	0.67
	Kawasaki ^c^	0.74	0.63–0.85	2755	0.76	0.65	0.42	0.42	0.78	0.69–0.88	2571	0.63	0.84	0.47	0.41
Na/Kratio ^d^		Criterion < 3.3 (*n* ^b^ = 39/80)								Criterion < 2.9 (*n* ^b^ = 57/122)					
1 time	-	0.74	0.63–0.85	3.7	0.56	0.80	0.37	0.48	0.79	0.71–0.87	2.7	0.82	0.68	0.50	0.37
2 times	-	0.82	0.73–0.91	3.4	0.74	0.80	0.55	0.32	0.87	0.80–0.93	3.2	0.77	0.88	0.65	0.26
3 times	-	0.84	0.75–0.93	3.4	0.85	0.78	0.63	0.27	0.87	0.81–0.93	3.1	0.82	0.83	0.66	0.24
4 times	-	0.84	0.75–0.92	3.4	0.79	0.78	0.58	0.30	0.89	0.83–0.94	3.1	0.82	0.82	0.64	0.25
5 times	-	0.86	0.78–0.94	3.2	0.85	0.76	0.60	0.29	0.90	0.84–0.95	3.2	0.81	0.88	0.68	0.23

Abbreviations: AUC, area under the curve; CI, confidence interval; ROC, receiver-operating characteristic; WFR, weighed food record; CO, cut-off value; Se, sensitivity; Spe, specificity; YI, Youden Index; DC, distance to corner; Kawasaki, estimated excretion values using Kawasaki equations; Na/K ratio, sodium-to-potassium ratio. ^a^ Cut-off values were determined from the Youden Index (sensitivity + specificity − 1) and distance to the corner {(1 − sensitivity)^2^ + (1 − specificity)^2^}; ^b^ number of participants who deviated from the criterion based on WFR as a reference measure; ^c^ 24-hour urinary potassium excretion and estimated 24-hour urinary potassium excretion based on the Kawasaki equation were adjusted to the intake level by multiplying by 1.3; ^d^ potassium intake based on the WFR used to calculate the Na/K ratio was adjusted to the 24-h urinary potassium excretion level by dividing by 1.3.

**Table 3:** AUC (95% CI) of the ROC curve of 24-hour urinary potassium excretion ^a^ to detect those with deviation from fruit and vegetable intake, measured based on the 12-day WFR.

	Men							Women						
	AUC	95% CI	CO ^b^	Se	Spe	YI	DC	AUC	95% CI	CO ^b^	Se	Spe	YI	DC
Fruit	Criterion ≥ 100 g (*n* ^c^ = 46/80)							Criterion ≥ 100 g (*n* ^c^ = 42/122)						
1 time	0.65	0.53–0.77	2867	0.61	0.74	0.34	0.47	0.68	0.58–0.78	2779	0.64	0.71	0.36	0.46
2 times	0.64	0.52–0.76	2697	0.52	0.74	0.26	0.55	0.68	0.58–0.77	2793	0.64	0.63	0.27	0.52
3 times	0.62	0.50–0.74	2576	0.41	0.85	0.27	0.61	0.71	0.62–0.81	2839	0.71	0.61	0.33	0.48
4 times	0.61	0.48–0.73	3030	0.63	0.56	0.19	0.58	0.69	0.59–0.79	3078	0.81	0.54	0.35	0.50
5 times	0.63	0.50–0.75	3024	0.67	0.53	0.20	0.57	0.68	0.58–0.78	2878	0.71	0.61	0.33	0.48
Vegetables	Criterion ≥ 350 g (*n* ^c^ = 44/80)							Criterion ≥ 350 g (*n* ^c^ = 70/122)						
1 time	0.77	0.66–0.87	3149	0.75	0.72	0.47	0.37	0.71	0.62–0.80	3348	0.86	0.52	0.38	0.50
2 times	0.80	0.70–0.90	3143	0.86	0.69	0.56	0.33	0.68	0.59–0.78	2988	0.70	0.63	0.33	0.47
3 times	0.77	0.67–0.88	3174	0.84	0.64	0.48	0.39	0.72	0.62–0.81	3085	0.77	0.62	0.39	0.45
4 times	0.76	0.65–0.88	3296	0.93	0.61	0.54	0.39	0.73	0.64–0.82	3073	0.77	0.69	0.46	0.38
5 times	0.77	0.66–0.88	3292	0.93	0.61	0.54	0.39	0.73	0.65–0.82	3000	0.71	0.69	0.41	0.42

Abbreviations: AUC, area under the curve; CI, confidence interval; ROC, receiver-operating characteristic; WFR, weighed food record; CO, cut-off value; Se, sensitivity; Spe, specificity; YI, Youden Index; DC, distance to corner. ^a^ The 24-hour urinary potassium excretion was adjusted to the intake level by multiplying by 1.3; ^b^ cut-off values were determined from the Youden Index (sensitivity + specificity − 1) and distance to the corner {(1 − sensitivity)^2^ + (1 − specificity)^2^}; ^c^ number of participants who deviated from the criterion based on the WFR as a reference measure.

**Table 4:** Number of days needed to assess mean values with 95% CIs within the specified % deviation of an individual’s mean from the usual (“true”) mean values identified based on the WFR or 24 h UC.

			Men (*n* = 80)						Women (*n* = 122)					
			Mean	CV_w_^a^	CV_b_^b^	Number of Days ^c^			Mean	CV_w_^a^	CV_b_^b^	Number of Days ^c^		
						±5%	±10%	±15%				±5%	±10%	±15%
Sodium	WFR (mg)	-	4561	28.7	23.1	32	8	4	3797	28.7	22.4	32	8	4
	24-h UC	Excretion (mg)	4674	31.0	23.0	37	10	5	3904	29.5	22.2	34	9	4
		Concentration (mEq/L)	122	26.2	27.7	27	7	3	109	24.0	26.4	23	6	3
		Kawasaki (mg)	5328	14.0	11.2	8	2	1	4832	13.4	12.0	7	2	1
Potassium	WFR (mg)	-	3175	24.8	28.8	24	6	3	2959	23.3	22.9	21	6	3
	24-h UC	Excretion (mg) ^d^	3008	25.3	27.9	25	7	3	3010	25.2	27.0	25	7	3
		Concentration (mEq/L)	35.9	30.5	25.7	36	9	4	38	23.7	24.3	22	6	3
		Kawasaki (mg) ^d^	2793	10.3	11.2	5	2	1	2775	10.7	12.7	5	2	1
Na/K ratio	WFR (mol/mol) ^e^	-	3.4	31.9	23.4	39	10	5	3.0	31.1	22.5	38	10	5
	24-h UC (mol/mol)	-	3.7	31.5	30.4	39	10	5	3.1	28.5	26.2	32	8	4
Fruit	WFR (g)	-	102	109.0	74.9	457	115	51	136	86.4	49.3	287	72	32
Vegetable	WFR (g)	-	370	47.8	44.6	88	22	10	349	42.7	32.3	71	18	8

Abbreviations: CIs, confidence intervals; WFR, weighed food record; 24-h UC, 24-hour urinary collection; CV_w_, coefficient of within-individual variation; CV_b_, coefficient of between-individual variation; Kawasaki, estimated excretion values using Kawasaki equations. ^a^ CV_w_ = {(within-individual variance)^0.5^/mean} × 100; ^b^ CV_b_ = {(between-individual variance)^0.5^/mean} × 100; ^c^ number of days needed to assess mean values with 95% CIs within the specified % deviation and the individual’s mean from usual mean values = (1.96 × CV_w_/specified % deviation)^2^; ^d^ 24-h urinary potassium excretion and estimated 24-h urinary potassium excretion values based on the Kawasaki equation were adjusted to the intake level by multiplying by 1.3; ^e^ potassium intake based on the WFR used to calculate the Na/K ratio was adjusted to the urinary excretion level by dividing by 1.3.

## Data Availability

In accordance with ethical guidelines in Japan aimed at ensuring participant privacy, individual data cannot be publicly disclosed. Furthermore, the informed consent we obtained did not include a provision for the data to be shared publicly. The datasets used and/or analyzed during the current study are available from the corresponding author on reasonable request.

## Supplementary Materials

The following supporting information can be downloaded at: https://www.mdpi.com/article/10.3390/nu16030442/s1, Table S1: AUC (95% CI) of ROC curves of one-time 24-h urinary sodium and potassium excretion and Na/K ratio in one-time 24-h urinary collection to detect those with deviating intakes of sodium, potassium, or Na/K measured by 12-day WFR using other criteria; Table S2: AUC (95% CI) of the ROC curves by one-time 24-h urinary potassium excretion to detect those with deviating intakes of fruit or vegetables measured by 12-day WFR using other criteria.
