# Supplementary material for: Urinary Biomarkers in Screening for the Usual Intake of Fruit and Vegetables, and Sodium, Potassium, and the Sodium-to-Potassium Ratio: Required Number and Accuracy of Measurements
Source: Nutrients. 2024 Feb 1;16(3):442. doi: 10.3390/nu16030442 (PMC10857367; doi:10.3390/nu16030442)
Supplement: Supplementary file 1 [file nutrients-16-00442-s001.zip › nutrients-2849187-supplementary.pdf]

Table S1. AUC (95% CI) of ROC curves of one-time 24-hour urinary sodium and potassium excretion and Na/K ratio in one-time 24-hour urinary collection to detect those with deviating intakes of sodium, potassium, or Na/K measured by 12-day WFR using other criteria

	**Men (n = 80)**								**Women (n = 122)**							
Criteria	n[a]	AUC	95% CI	CO[b]	Se	Spe	YI	DC	n[a]	AUC	95% CI	CO[b]	Se	Spe	YI	DC
**Sodium** (in salt equivalent, g)																
<7.0 g	77	*0.79*	*0.63 - 0.95*	3105	0.79	0.67	0.46	0.39	112	*0.84*	*0.75 - 0.93*	3074	0.78	0.80	0.58	0.30
<8.0 g	72	*0.74*	*0.51 - 0.97*	3621	0.68	0.75	0.43	0.41	92	*0.79*	*0.71 - 0.88*	3657	0.65	0.83	0.49	0.39
<9.0 g	65	*0.76*	*0.60 - 0.92*	3621	0.72	0.73	0.46	0.38	72	*0.75*	*0.66 - 0.83*	3988	0.61	0.80	0.41	0.44
<10.0 g	55	*0.79*	*0.67 - 0.90*	4163	0.71	0.80	0.51	0.35	48	*0.72*	*0.63 - 0.82*	3996	0.67	0.73	0.40	0.43
<11.0 g	46	*0.75*	*0.64 - 0.86*	4255	0.70	0.71	0.40	0.42	28	*0.70*	*0.59 - 0.81*	4048	0.64	0.69	0.33	0.47
<12.0 g	30	*0.70*	*0.59 - 0.82*	4255	0.77	0.62	0.39	0.45	18	*0.76*	*0.63 - 0.89*	4674	0.67	0.82	0.48	0.38
<13.0 g	23	0.68	0.56 - 0.81	4255	0.74	0.56	0.30	0.51	10	*0.73*	*0.58 - 0.87*	3996	0.80	0.61	0.41	0.44
<14.0 g	16	*0.71*	*0.56 - 0.85*	4444	0.75	0.59	0.34	0.48	5	0.63	0.38 - 0.88	5351	0.40	0.88	0.28	0.61
<15.0 g	9	*0.70*	*0.50 - 0.89*	6074	0.56	0.82	0.37	0.48	3	0.76	0.44 - 1.00	5351	0.67	0.88	0.55	0.35
**Potassium**[c]																
≥2000 mg	7	*0.82*	*0.66 - 0.99*	1994	0.71	0.86	0.58	0.32	7	*0.80*	*0.67 - 0.92*	2814	1.00	0.60	0.60	0.40
≥2500 mg	19	*0.85*	*0.77 - 0.94*	2679	0.84	0.72	0.56	0.32	36	*0.78*	*0.70 - 0.87*	2727	0.69	0.73	0.43	0.41
≥3000 mg	34	*0.77*	*0.67 - 0.88*	2814	0.74	0.78	0.52	0.34	66	*0.73*	*0.64 - 0.82*	3190	0.77	0.59	0.36	0.47
≥3500 mg	53	*0.79*	*0.69 - 0.90*	3091	0.72	0.85	0.57	0.32	93	*0.82*	*0.73 - 0.91*	3441	0.87	0.72	0.60	0.30
≥4000 mg	69	0.69	0.51 - 0.86	3149	0.59	0.82	0.41	0.44	113	*0.85*	*0.75 - 0.95*	3569	0.82	0.89	0.71	0.21
**Na/K ratio**[d]																
<2.0	77	*0.97*	*0.93 - 1.00*	1.8	0.97	0.67	0.64	0.33	116	*0.94*	*0.89 - 0.98*	1.8	0.92	0.83	0.76	0.18
<2.5	64	*0.84*	*0.74 - 0.94*	2.7	0.80	0.69	0.48	0.37	86	*0.84*	*0.77 - 0.91*	2.8	0.72	0.89	0.61	0.30
<3.0	53	*0.79*	*0.70 - 0.89*	3.2	0.70	0.78	0.48	0.37	48	*0.78*	*0.69 - 0.86*	2.8	0.83	0.65	0.48	0.39
<3.5	29	*0.74*	*0.63 - 0.86*	4.4	0.55	0.92	0.47	0.46	20	*0.79*	*0.70 - 0.87*	3.5	0.75	0.75	0.50	0.35
<4.0	14	*0.90*	*0.80 - 1.00*	4.4	0.93	0.89	0.82	0.13	10	*0.84*	*0.75 - 0.93*	3.5	0.90	0.75	0.65	0.27
<4.5	8	*0.81*	*0.65 - 0.97*	4.4	0.88	0.82	0.69	0.22	3	*0.96*	*0.92 - 1.00*	5.0	1.00	0.95	0.95	0.05

Abbreviations: AUC, area under the curve; CI, confidence interval; ROC, receiver-operating characteristics; WFR, weighed food records; CO, cut-off values; Se, sensitivity; Spe, specificity; YI, Youden's Index; DC, Distance to corner; Na/K ratio, sodium-to-potassium ratio.

Italic figures mean that the cumulative values of two or more measurements were also shown to be useful.

[a] Number of participants who deviated from the criterion based on WFR as reference measure; [b] Cut-off values were determined from Youden Index (sensitivity + specificity − 1) and distance to corner $\{(1 - \text{sensitivity})^2 + (1 - \text{specificity})^2\}$; [c] 24-hour urinary potassium excretion was adjusted to the intake level by multiplying by 1.3; [d] Potassium intake by WFR used to calculate Na/K ratio, adjusted to the 24-hour urinary potassium excretion level by dividing by 1.3.

Table S2. AUC (95% CI) of the ROC curves by one-time 24-h urinary potassium excretion[a] to detect those with deviating intakes of fruit or vegetables measured by 12-day WFR using other criteria

	Men (n=80)								Women (n=122)							
Criteria	n[b]	AUC	95% CI	CO[c]	Se	Spe	YI	DC	n[b]	AUC	95% CI	CO[c]	Se	Spe	YI	DC
**Fruit**																
≥ 50 g	29	0.59	0.46 - 0.73	2867	0.62	0.63	0.25	0.53	18	*0.73*	*0.60 - 0.85*	2727	0.78	0.67	0.45	0.40
≥100 g	46	0.65	0.53 - 0.77	2867	0.61	0.74	0.34	0.47	42	0.68	0.58 - 0.78	2779	0.64	0.71	0.36	0.46
≥150 g	60	0.62	0.48 - 0.75	2867	0.53	0.75	0.28	0.53	69	0.68	0.59 - 0.78	2867	0.59	0.75	0.35	0.47
≥200 g	70	0.61	0.41 - 0.80	3091	0.56	0.70	0.26	0.53	97	0.69	0.57 - 0.80	3353	0.77	0.56	0.33	0.50
≥250 g	76	0.64	0.27 - 1.00	4213	0.91	0.50	0.41	0.51	116	0.62	0.36 - 0.88	3353	0.72	0.67	0.39	0.43
**Vegetables**																
≥200 g	8	*0.79*	*0.65 - 0.93*	2601	0.88	0.68	0.56	0.34	11	0.78	0.67 - 0.89	2814	0.91	0.61	0.52	0.40
≥250 g	23	*0.77*	*0.65 - 0.90*	2601	0.74	0.77	0.51	0.35	26	0.71	0.61 - 0.82	2903	0.73	0.63	0.36	0.46
≥300 g	28	*0.79*	*0.68 - 0.90*	2626	0.75	0.81	0.56	0.32	48	0.71	0.62 - 0.81	2903	0.67	0.69	0.36	0.46
≥350 g	44	*0.77*	*0.66 - 0.87*	3149	0.75	0.72	0.47	0.37	70	0.71	0.62 - 0.80	3348	0.86	0.52	0.38	0.50
≥400 g	51	*0.72*	*0.60 - 0.83*	2837	0.59	0.79	0.38	0.46	84	*0.74*	*0.64 - 0.84*	3348	0.83	0.61	0.44	0.43
≥450 g	60	0.69	0.55 - 0.83	3207	0.63	0.70	0.33	0.47	97	*0.75*	*0.64 - 0.87*	3348	0.79	0.68	0.47	0.38
≥500 g	67	*0.75*	*0.59 - 0.92*	3207	0.63	0.85	0.47	0.40	108	*0.86*	*0.73 - 0.98*	3466	0.82	0.86	0.68	0.23
≥550 g	70	0.69	0.50 - 0.88	3207	0.60	0.80	0.40	0.45	116	*0.92*	*0.84 - 1.00*	3680	0.82	0.83	0.65	0.25
**Combined**																
≥250 g	10	*0.80*	*0.69 - 0.92*	2434	0.80	0.79	0.59	0.29	11	0.73	0.61 - 0.85	2814	0.82	0.60	0.42	0.44
≥300 g	16	*0.87*	*0.78 - 0.95*	2601	0.94	0.77	0.70	0.24	15	*0.77*	*0.67 - 0.87*	2814	0.87	0.63	0.49	0.40
≥350 g	21	*0.83*	*0.72 - 0.94*	2626	0.86	0.78	0.64	0.26	22	*0.79*	*0.70 - 0.88*	2727	0.82	0.70	0.52	0.35
≥400 g	34	*0.77*	*0.66 - 0.88*	2679	0.68	0.78	0.46	0.39	41	*0.81*	*0.73 - 0.89*	2727	0.76	0.79	0.55	0.32
≥450 g	41	*0.70*	*0.58 - 0.81*	2837	0.63	0.74	0.38	0.45	54	*0.77*	*0.69 - 0.85*	2928	0.72	0.71	0.43	0.40
≥500 g	51	0.66	0.54 - 0.78	2867	0.59	0.76	0.35	0.48	66	*0.75*	*0.66 - 0.83*	3348	0.91	0.55	0.46	0.46
≥550 g	57	*0.72*	*0.59 - 0.84*	2979	0.63	0.83	0.46	0.41	83	*0.70*	*0.59 - 0.80*	3353	0.83	0.56	0.40	0.47
≥600 g	64	*0.75*	*0.61 - 0.88*	3149	0.64	0.88	0.52	0.38	94	*0.75*	*0.63 - 0.87*	3353	0.83	0.71	0.54	0.33
≥650 g	66	*0.72*	*0.57 - 0.87*	3149	0.62	0.86	0.48	0.40	106	*0.82*	*0.69 - 0.95*	3466	0.83	0.81	0.64	0.25
≥700 g	70	0.68	0.49 - 0.88	3149	0.59	0.80	0.39	0.46	110	*0.87*	*0.76 - 0.99*	3569	0.84	0.83	0.67	0.23
≥750 g	72	0.66	0.43 - 0.90	3530	0.75	0.63	0.38	0.45	116	0.80	0.62 - 0.97	3569	0.80	0.83	0.64	0.26
≥800 g	75	0.76	0.45 - 1.00	3904	0.85	0.80	0.65	0.25	118	0.76	0.50 - 1.00	3569	0.79	0.75	0.54	0.33

Abbreviations: AUC, area under the curve; CI, confidence interval; ROC, receiver-operating characteristics; WFR, weighed food records; CO, cut-off values; Se, sensitivity; Spe, specificity; YI, Youden's Index; DC, Distance to corner.

Italic figures mean that the cumulative values of two or more times also showed usefulness.

[a] 24-hour urinary potassium excretion was adjusted to the intake level by multiplying by 1.3; [b] Number of participants who deviated from the criterion based on WFR as reference measure; [c] Cut-off values were determined from Youden Index (sensitivity + specificity − 1) and distance to corner $\{(1 - \text{sensitivity})^2 + (1 - \text{specificity})^2\}$.
